# A Closer Examination of the ‘Abundant-Center’ for Ectomycorrhizal Fungal Community Associated With *Picea crassifolia* in China

**DOI:** 10.3389/fpls.2022.759801

**Published:** 2022-02-24

**Authors:** Xiaobing Wang, Qisheng Han

**Affiliations:** ^1^School of Civil Engineering and Architecture, Xinxiang University, Xinxiang, China; ^2^Farmland Irrigation Research Institute, Chinese Academy of Agricultural Sciences, Xinxiang, China

**Keywords:** ectomycorrhizal fungi, abundance-centre hypothesis, *Picea crassifolia*, richness, community structure

## Abstract

A long-standing hypothesis in biogeography predicts that a species’ abundance is highest at the center of its geographical range and decreases toward its edges. In this study, we test the *abundant-center hypothesis* of ectomycorrhizal (ECM) fungal communities associated with *Picea crassifolia*, an endemic species widely distributed in northwest China. We analyzed the taxonomic richness and the relative abundance of ECM fungi in four main distribution areas, from center to edges. In total, 234 species of ECM fungi were detected, and of these, 137 species were shared among all four sites. *Inocybe*, *Sebacina*, *Tomentella*, and *Cortinarius* were the dominant genera. ECM fungal richness and biodiversity were highest at the central and lower at peripheral sites. Our results indicated that ECM fungal species richness was consistent with the *abundant-center hypothesis*, while the relative abundances of individual fungal genera shifted inconsistently across the plant’s range.

## Introduction

Ectomycorrhiza (ECM) is an intimate symbiotic association between plant roots and ECM fungi. ECM associations benefit most terrestrial plants by enhancing nutrient uptake and tolerance to environmental stressors (Tedersoo et al., 2020). ECM fungal community structure is affected by a multitude of biotic and abiotic factors (Ishida et al., 2007). The community composition is shown to change across gradients of soil pH (Bahram et al., 2011), soil nitrogen and organic matter (Pellitier et al., 2021), elevation (Matsuoka et al., 2016), and forest age (Hui et al., 2017; Boeraeve et al., 2018). As biotic and abiotic components usually show strong interdependencies, it is methodologically difficult to assess the contributions made by each specific factor in the ECM fungal community and their response to environmental changes (Weißbecker et al., 2018).

The effect of different driving factors at different scales is variable. Physiographic factors such as altitude and soil properties including soil microorganisms and soil physicochemical properties are usually important drivers of ECM fungal communities in the local-scale sampling area, such as carbon content and pH (Vasco-Palacios et al., 2020), altitude and forest age (Matsuoka et al., 2016; Geml et al., 2017; Schön et al., 2018) and host genetic and phenotypic characteristics (Rúa, 2021). In large-scale ECM fungal studies, the role of host plants is becoming increasingly obvious. Host dispersal can affect the biogeographic patterns of the ECM fungal community (Matsuoka et al., 2019), and a significant proportion of ECM fungi exhibited host specificity in conifer broadleaf forests (Ishida et al., 2007). In addition, ECM fungal communities show a stronger correlation with host composition than soil properties, while pathogens and arbuscular mycorrhizal (AM) fungi are more strongly correlated with soil properties (Schappe et al., 2020). Hence, whether the range of host habitats is the main factor for ECM fungal community structure is worth further exploring.

The *abundant-center hypothesis* posits that species abundance peaks in the center of its distributional range and declines toward its edges (Hengeveld and Haeck, 1982). The high abundance in the center is because of the optimal conditions (i.e., the presence of suitable habitats), while the decline in abundance at edges is because of environmental suboptimality (Lira-Noriega and Manthey, 2014). A recent modeling study showed that the *abundant-center* relationships were strongly affected by populations and deterministic growth environment (Dallas and Santini, 2020). This theory provides an overview of species distributions. However, studies on *abundant-center hypothesis* have mainly focused on animals and plants (Pironon et al., 2017), while studies on ECM fungal communities are rare.

Unlike the animal and plant species, ECM fungi are typical symbionts and more than 90% of nutrient-absorbing roots of trees are colonized by ECM fungi (Smith and Smith, 2002). ECM fungi are estimated to comprise 20,000–25,000 species globally and form symbioses with only a tiny fraction of the terrestrial plants (2% of total plant species) (Tedersoo et al., 2012). In addition, most ECM fungi reproduce sexually and produce macroscopic fruit bodies, which differ from arbuscular and ericoid mycorrhizas (Tedersoo et al., 2010). Because these fruit bodies deposit most of their spores within in a short distance, the spatial distribution of the ECM community is probably more dependent on the host plants’ distribution than environmental factors. Hence, the distribution models between ECM fungi and host plants may be the same, or at least very similar. Most studies have been performed on the relationship between host plant species composition and ECM fungal community (Durall et al., 2006; Mucha et al., 2018), while fewer studies have addressed the effects of host plants’ abundance. The distribution of some Pinaceae species, which is certainly the oldest extant plant family symbiotic with ECM fungi (Hibbett and Matheny, 2009), have found evidence for the *abundant-center hypothesis* (Mimura and Aitken, 2007; Gugerli et al., 2009). Previous studies have found that ECM communities are more concentrated on species of Pinaceae and showed stronger host effects than on broadleaf species (Ishida et al., 2007; Vlk et al., 2020). However, for ECM fungal distribution modeling, it appears important to consider not only the distribution of individual species but also the ECM fungal richness and biodiversity of the whole community. Many ECM fungi may fulfill similar ecological functions and that degree of functional redundancy exists in ECM fungal communities (Dahlberg, 2001). ECM fungi are usually categorized into two major groups based on their distribution features: cosmopolitan ECM members (*Cortinarius*, *Russula* and *Tomentella*) and endemic ECM members (McPolin and Kranabetter, 2021). Endemic ECM fungi usually exhibit higher host specialization than cosmopolitan ECM members, which is generally driven by the host range (McPolin and Kranabetter, 2021). *Picea crassifolia* is one of the main tree species that belongs to the Pinaceae family in semi-arid areas of China. It forms pure stands on north-facing slopes along the northeast edge of the Qinghai-Tibetan Plateau (QTP) region and in the adjacent highlands of Helan Mountain, which has not been subject to the significant human disturbance for more than one century. The ECM fungal community associated with a single host in the pure forest of *P. crassifolia* was considered an excellent material for ecological studies.

In this study, we examined the ECM fungal community associated with *P. crassifolia* to test if the distribution of ECM fungal species fit the *abundant-center hypothesis* model. We set out two hypotheses: (1) Is the composition of ECM fungal communities associated with *P. crassifolia* generally consistent across the host’s geographical range? (2) Does the richness and diversity of these ECM fungi decrease from the center to the edge of the host’s distribution?

## Materials and Methods

### Study Area and Experimental Manipulation

**FIGURE 1:**
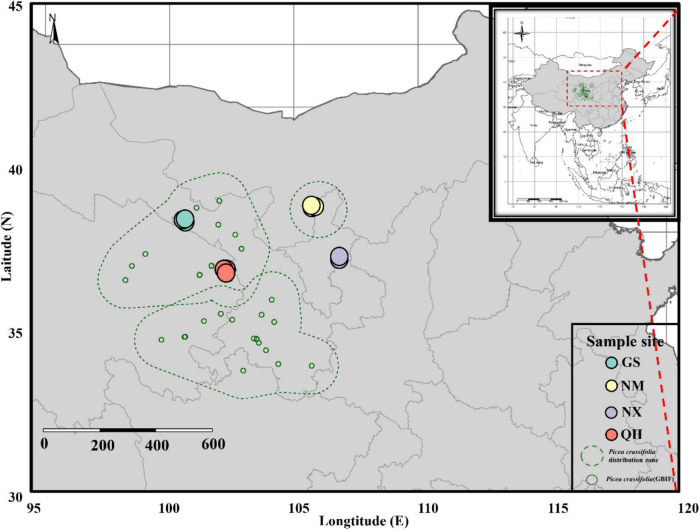
Map of sampling site locations in China.

*Picea crassifolia* is mainly distributed on the north-facing slopes along the northeast edge of the QTP region and in the adjacent highlands of Helan Mountain. To show the distribution map of the *P. crassifolia* directly, we compiled occurrence data from the GBIF database^1^ and maps were generated using the *tmap* (Tennekes et al., 2021) and *rangemap* (Cobos et al., 2021) packages in R. Field sampling was conducted in four pure *P. crassifolia* stands in Qinghai (QH), Gansu (GS), Ningxia (NX), and Mongolia (NM) provinces as representative of its complete distribution. As shown in Figure 1, *P. crassifolia* has a higher abundance in QH sites (core site) and a lower abundance in NX, NM and GS sites (edge sites). In addition, the geographical distances between the core-edge QH-GS, QH-NX, and QH-NM are approximately 242, 351, and 364 km, respectively. These forests are estimated to be aged above 70 years.

Three sampling plots were randomly located in each forest stand in September 2016. At each plot, 10 spruce trees, spaced 10 m apart to avoid possible spatial autocorrelation (Lilleskov et al., 2004; Peay et al., 2010b), were selected for sampling. For each tree, three fine roots (approximately 10 cm in length) were collected within 1 m from the trunk. The collected root samples for each plot were put in a single plastic bag and stored on ice in a cooling container. Soil samples were collected from the same sampling plot. All samples were stored in the laboratory at 4°C before being processed but were not stored longer than 1 week before processing.

### Soil Analyses

After removing visible plant material, parts of soil samples were dried and passed through a 2 mm sieve to determine pH and electric conductivity (EC), while other parts were further sieved through a 250 μm screen to determine soil organic carbon (SOC), total nitrogen (TN) and total phosphorus (TP). The soil pH and EC values were, respectively, measured using a Sartorius PB-10 pH meter (Sartorius Co., Ltd., Göttingen, Germany) and conductivity meter (DDS-307, Shanghai REX Instrument Factory, China) by mixing soil with distilled water (1:2). The SOC was determined using the Walkley-Black (WB) method by quantifying the amount of oxidizable soil carbon with a reaction with acidic dichromate (Cr^2^O_7_^2–^). The TN content was determined by the semimicro Kjeldahl method (AutoAnalyzer 3, Bran + luebbe, Hamburg, Germany). The TP content was determined by the colorimetric method using the application of acid-soluble molybdenum antimony anti-determination.

### DNA Extraction and Illumina Sequencing

Total genome DNA from the root sample was extracted using the cetyltrimethylammonium bromide (CTAB) method. The concentration and purity of DNA were monitored on 1% agarose gels. According to the concentration, DNA was diluted to 1 ng/μl using sterile water. For PCR reactions, the internal transcribed spacer (ITS) genes of distinct regions were amplified using ITS1/ITS2 primers (Sequence: TCCGTAGGTGAACCTGCGG/GCTGCGTTCTTCATCGATGC) with the barcode (Bengtsson-Palme et al., 2013). All PCR reactions were carried out using Phusion^®^ High-Fidelity PCR Master Mix (New England Biolabs), 2 μM of forward and reverse primers, and approximately 10 ng template DNA. Thermal cycling consisted of initial denaturation at 98°C for 1 min, followed by 30 cycles of denaturation at 98°C for 10 s, annealing at 50°C for 30 s and elongation at 72°C for 30 s; finally, 72°C for 5 min. Mix same volume of 1 × loading buffer (containing SYB green) with PCR products and operate electrophoresis on 2% agarose gel for detection. Then, mixed PCR products were purified using a Qiagen Gel Extraction Kit (Qiagen, Germany). Sequencing libraries were generated using a TruSeq^®^ DNA PCR-Free Sample Preparation Kit (Illumina, San Diego, CA, United States), following the manufacturer’s recommendations and adding index codes. The library quality was assessed using a Qubit^®^ 2.0 Fluorometer (Thermo Scientific, Landsmeer, Netherlands) and Agilent Bioanalyzer 2100 system. As a final step, the library was sequenced using an Illumina Hiseq 2500 platform, and 250 bp paired-end reads were generated. Paired-end reads were assigned to samples based on their unique barcode and truncated by cutting off the barcode and primer sequence. Reads were then merged using FLASH (version 1.2.7^2^) (Magoč and Salzberg, 2011), which was designed to merge paired-end reads when at least some of the reads overlap the reads generated from the opposite end of the same DNA fragment. Spliced sequences were called raw tags.

### Bioinformatic Analyses

Quality filtering on the raw tags was performed under specific filtering conditions to obtain the high-quality clean tags (Bokulich et al., 2013) using the quantitative insights into microbial ecology (QIIME) (version 1.7.0^3^) quality-controlled process (Caporaso et al., 2010). Tags were compared with a reference database (Gold database^4^) using the UCHIME algorithm^5^ to identify chimeric sequences. When detected, chimeric sequences were removed and effective tags were obtained. Sequence analyses were performed using the Uparse software (Uparse version 7.0.1001^6^) (Edgar, 2013). Sequences with ≥97% of similarity were assigned to the same operational taxonomic unit (OTUs). OTUs represented by <10 reads were discarded as sequencing errors (Nguyen et al., 2015).

The representative sequence for each OTU was individually checked using basic local alignment search tool (BLAST) with the NCBI database^7^ and UNITE database^8^. Sequences identified as originating from non-ECM taxa (Tedersoo et al., 2010) were removed from the dataset to restrict analyses to taxa with evidence of ECM status. As mentioned earlier, we used the relative abundance of ECM fungal species instead of the absolute abundance values as the abundance index for all analyses. All the sequences reported in this study have been submitted to the DDBJ database (LC203765- LC205656).

### Statistical Analyses

Chao2 index, Shannon’s diversity index and Simpson’s diversity index were used to characterize OTU sample diversity at each site, and they were calculated using *Esimate*S (Colwell, 2021). Statistical comparisons of soil data and biodiversity indices among different samples were made with a one-way analysis of variance (ANOVA) using R (R Core Team, 2021). Analysis of similarity (ANOSIM) was used for site difference test between community groups. To statistically assess the effect of the environmental factors on the ECM composition, a multivariate analysis of variance based on dissimilarity test (ADONIS) was applied using the vegan package in R software (Oksanen, 2021). Redundancy analysis (RDA) between the relative abundance of ECM fungi and environmental factors was performed to explore the main factors determining the ECM community structure. For RDA analyses, data were log + 1 transformed. Co-linearity between environmental variables was checked using variance inflation factors (VIF), and the environmental variables with VIF > 10 were excluded from the RDA analyses. The model ran for 1,000 permutations and the significance of the explanatory variables and the first two RDA axes were assessed using ANOVA. Isolation by distance was tested through the correlation between geographical distances (calculated from GPS coordinates) and Bray–Curtis distances using a Mantel test (Smouse et al., 1986). Because of the significant relationship between species composition and distance for each sample site, we calculated a Mantel correlogram to test the strength of correlation at different distance classes by using Ecodist package (Peay et al., 2010a). To test whether ECM fungal species richness changed from the core to the edge, we modeled decrease rates with geographical distances from core to edge:

Decrease rate = (Highest observed richness in core site – the observed richness in other sites)/(Highest observed richness in core site).

To improve the normality of residuals, the geographical distance between any two points was log10 transformed. Linear discriminant analysis (LDA) effect size (LefSe) analysis was performed to investigate the differences in the ECM fungal relative abundance among all groups with the *microeco* statistical package (Liu et al., 2021).

## Results

### Soil Characteristics

Variation in soil pH among the four investigated stands was small, but the differences were significant at four sites (Table 1). The changes in the soil SOC content coincided well with those of the soil TN content; NM was the lowest (59.14 ± 5.71, 2.86 ± 0.16) and QH was the highest (129.68 ± 37.15, 5.9 ± 0.92). However, no obvious variation in the soil TP content was observed among sites (Table 1).

**TABLE 1:** Summary of soil characteristics recorded of the study stands.

Site	NM	NX	GS	QH
pH	7.75 ± 0.11 ab	7.94 ± 0.02 b	7.61 ± 0.08 a	7.65 ± 0.05 a
EC (μS/cm)	198.67 ± 53.11 ab	136 ± 3.61 a	343 ± 112.79 b	354.67 ± 87.93 b
SOC (g⋅kg^–1^)	59.14 ± 5.71 a	64.4 ± 7.38 a	68.51 ± 23.35 a	129.68 ± 37.15 b
TN (g⋅kg^–1^)	2.86 ± 0.16 a	3.04 ± 0.28 a	4.46 ± 1.1 ab	5.9 ± 0.92 b
TP (g⋅kg^–1^)	0.62 ± 0.06	0.74 ± 0.01	0.73 ± 0.04	0.68 ± 0.07

*Mean values in the raw differ significantly after (p < 0.05) if they have no letter (a and b) in common (ANOVA tests followed by a Tukey’s test).*

### Ectomycorrhizal Community Description

**FIGURE 2:**
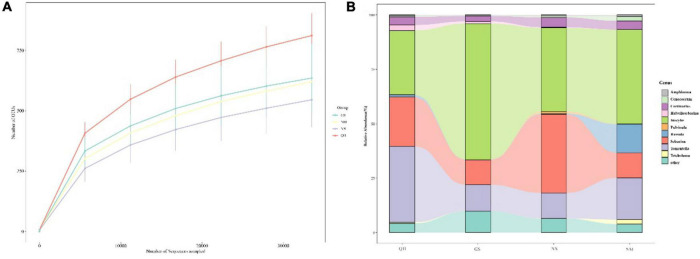
(A) Rarefaction curves of OTU number at 97% of similarity for each site. Mean values of three replicates and the error bar are shown. (B) Alluvial diagram of the top 10 genera’s relative abundance in four sites.

In total, 755,738 sequence reads were generated using Illumina Hiseq 2500. The average length of the effective tags was 233.9 bq. Rarefaction curves in all sites (Figure 2A) indicate that sequencing depth can reveal fungal community composition. After removing the sequence reads that did not match ECM fungal taxa, 384,558 sequences (50.8% of total sequences) were left for analyses and interpretation. Of these, we removed 378 that possessed <10 reads. In total, 234 ECM OTUs were identified (Supplementary File 1).

Most of the ECM OTUs (93.6%) belonged to the phylum Basidiomycota, while Ascomycota only accounted for a small fraction (14 OTUs). In total, OTUs were clustered into overall 27 ECM fungal families. Among these families, Inocybaceae, Sebacinaceae, and Thelephoraceae had a higher relative abundance (43.8, 21.3, and 21.05%, respectively). Among them, 87% of the ECM OTUs could be accurately assigned at the genus level. Major ECM fungal genera in this study included the following: *Inocybe* (56 species), *Tomentella* (46 species), *Cortinarius* (22 species), and *Sebacina* (20 species). At the species level, *Inocybe* sp.1 was the most abundant fungal taxon, with recorded reads, followed by *I*nocybe *flocculosa* and *Sebacina* sp.9.

The distribution of relative abundances among these ECM genera was similar among the NX, NM, QH, and GS sites. However, *Inocybe*, *Sebacina*, *Tomentella*, and *Cortinarius* were the top four genera at the NX, QH, and GS sites, while the *Russula* replaced the *Cortinarius* as the dominant genus at the NM site. At all of the sampled edge sites (GS, NM, and NX), the fungal genus *Inocybe* had the highest relative abundance, while *Tomentella* was more dominant than *Inocybe* at the center site (QH) (Figure 2B).

**FIGURE 3:**
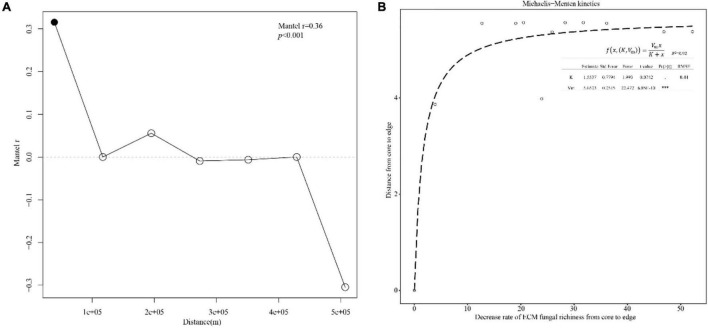
**(A)** Spatial correlogram between Mantel statistics and distance class index: significant Mantel correlation (black points) indicates spatial correlation. **(B)** Decrease rate of ECM fungal richness from center to edges. The data were fitted to Michaelis–Menten kinetics, and the dashed line shows a significant non-linear regression.

There was a significant spatial autocorrelation between ECM fungal communities (*P* < 0.001), a Mantel correlogram suggested spatial autocorrelation only within ∼39 km (Figure 3A). The center site (QH) has the highest number of observed ECM richness and mean richness per site. The decrease rates of ECM fungal richness with distances followed Michaelis–Menten kinetics and the regression equation model has a good goodness of fit. The parameters for the model are shown in Figure 3B. The result showed that the richness value fell by 10% or more from core to edge. Estimated richness and diversity (Shannon’s and Simpson’s index) showed the same trends (Table 2). ECM fungal communities were dissimilar between sites (*R* = 0.33; *P* = 0.006, Anosim).

**TABLE 2:** Summary of ectomycorrhizal fungal diversity associated with *P. crassifolia.*

Site	NM (edge)	NX (edge)	GS (edge)	QH (center)
Mean richness per site	148 ± 24.98^ab^	139.67 ± 11.5^ab^	109.33 ± 24.58^a^	168.67 ± 19.22^b^
Observed richness	196	186	167	211
Estimated richness (Chao2)	202 ± 17.5	200 ± 17.6	196 ± 25.6	222 ± 16.1
Shannon’s diversity index	5.12 ± 0.13^ab^	5.06 ± 0.11^ab^	4.87 ± 0.18^a^	5.22 ± 0.09^b^
Simpson’s diversity index	163.92 ± 15.18^b^	153.59 ± 12.21^ab^	126.83 ± 16.44^a^	180.94 ± 11.67^b^

*Values are means ± SE (n = 3). ^a,b^Significantly different (P < 0.05) by ANOVA.*

**FIGURE 4:**
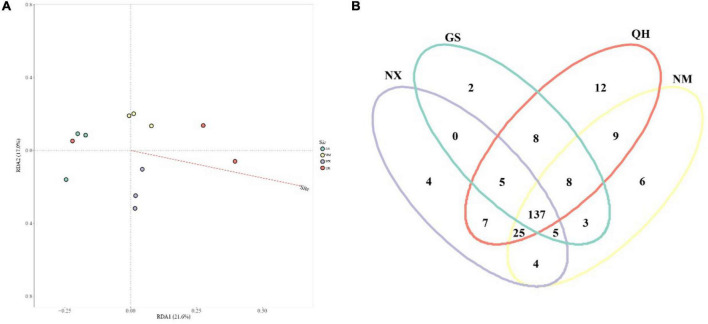
**(A)** Redundancy analysis (RDA) plot of ECM fungal community and environmental variables among four sites at the species level. The percentage of the *X*-axis and *Y*-axis represents the proportion of explained variance. **(B)** Venn diagram illustrating the number of unique and shared operational taxonomic units.

However, the site was the strongest factor associated with compositional variation (*P* < 0.01, *R*^2^ = 0.37, Adonis). Direct gradient ordination of ECM fungal communities using RDA showed that the effect of site was highly significant (*P* < 0.001) in explaining the distribution of ECM species (Figure 4A). Shared species analysis showed that of a total of 234 OTUs, 137 were shared among all four sites (Figure 4B). We compared the values of the relative abundance at genus level and nearly half of ECM fungal genera (18) were higher at the center site than that at the edge sites (Table 3).

**TABLE 3:** Comparing the differences of the ECM fungal relative abundance at genus level in four sites.

Genus	QH	GS	NX	NM	(Center > edge)
*Amanita*	0.19 ± 0.22*^a^*	0.06 ± 0.03*^b^*	0 ± 0*^b^*	0.01 ± 0.01*^b^*	1
*Amphinema*	0.57 ± 0.38	0.28 ± 0.25	0.47 ± 0.54	0.62 ± 0.83	0
*Cenococcum*	0.58 ± 0.28	0.18 ± 0.28	0.47 ± 0.81	2.18 ± 2.62	0
*Ceratobasidium*	0.17 ± 0.23	0.08 ± 0.1	0.1 ± 0.05	0.19 ± 0.14	0
*Choiromyces*	0.33 ± 0.50	0.02 ± 0.01	0.49 ± 0.6	0.19 ± 0.3	0
*Chroogomphus*	0.03 ± 0.03	0 ± 0	0 ± 0	0 ± 0	1
*Clavulina*	0.008 ± 0.01	0 ± 0	0 ± 0	0.006 ± 0.01	1
*Clitocybe*	0.013 ± 0.02a	0 ± 0	0.008 ± 0.01b	0 ± 0	1
*Clitopilus*	0 ± 0	0.01 ± 0.01	0 ± 0	0 ± 0	0
*Cortinarius*	4.37 ± 2.22	4.66 ± 4.47	7.11 ± 11.16	3.69 ± 5.81	0
*Entoloma*	0.19 ± 0.08	0.05 ± 0.08	0.02 ± 0.01	0.01 ± 0.01	1
*Geopora*	0.01 ± 0.01	0 ± 0	0 ± 0	0.02 ± 0.04	0
*Gomphidius*	0.02 ± 0.04	0 ± 0	0 ± 0	0 ± 0	1
*Hebeloma*	0.06 ± 0.02	0.53 ± 0.92	0.02 ± 0.01	0.12 ± 0.18	0
*Helvellosebacina*	1.8 ± 2.76*^a^*	0.61 ± 0.91*^b^*	0.21 ± 0.36*^b^*	0.01 ± 0*^b^*	1
*Inocybe*	29.71 ± 7.05	47.26 ± 31.68	39.33 ± 10.16	45.12 ± 31.66	0
*Lactarius*	0.11 ± 0.12	0.03 ± 0.04	0.59 ± 0.96	0.04 ± 0.06	0
*Lyophyllum*	0.51 ± 0.84*^a^*	0.06 ± 0.10*^b^*	0.06 ± 0.1*^b^*	0 ± 0	1
*Melanogaster*	0.04 ± 0.03*^a^*	0 ± 0	0 ± 0	0.01 ± 0.01*^b^*	1
*Orbilia*	0.011 ± 0.01*^a^*	0 ± 0	0.007 ± 0.01*^b^*	0 ± 0	1
*Peziza*	0 ± 0	0.07 ± 0.10	0.03 ± 0.04	0.01 ± 0.01	0
*Phellodon*	0.04 ± 0.07	0 ± 0	0 ± 0	0 ± 0	1
*Piloderma*	0.02 ± 0.01	0.02 ± 0.02	1.63 ± 2.77	0.01 ± 0.01	0
*Pseudotomentella*	0.27 ± 0.22*^a^*	0 ± 0	0.01 ± 0.01*^b^*	0.21 ± 0.34*^a^*	1
*Pulvinula*	0.05 ± 0.02	0.03 ± 0.02	0.78 ± 1.28	0.09 ± 0.11	0
*Pustularia*	0.014 ± 0.01*^a^*	0.007 ± 0.01*^b^*	0.009 ± 0.01*^b^*	0 ± 0	1
*Russula*	1.24 ± 0.71	0.17 ± 0.29	0.36 ± 0.21	12.11 ± 20.63	0
*Sarcosphaera*	0.02 ± 0.02	0.01 ± 0.01	0 ± 0	0 ± 0	1
*Sebacina*	25.63 ± 8.64	23.63 ± 32.79	29.54 ± 27.35	11.39 ± 6.82	0
*Serendipita*	0.14 ± 0.18*^a^*	0.02 ± 0.01*^b^*	0.01 ± 0.01*^b^*	0.01 ± 0.01*^b^*	1
*Sistotrema*	0.02 ± 0.01	0 ± 0	0.1 ± 0.17	0.01 ± 0.01	1
*Suillus*	0.52 ± 0.55*^a^*	0.01 ± 0.01*^b^*	0.02 ± 0.03*^b^*	0.08 ± 0.11*^b^*	1
*Thelephora*	0.02 ± 0.02	0.01 ± 0.02	0.05 ± 0.08	0 ± 0	0
*Tomentella*	30.44 ± 13.76	12.94 ± 8.31	11.65 ± 0.68	18.64 ± 10.05	1
*Tomentellopsis*	0 ± 0	0 ± 0	0.02 ± 0.04	0 ± 0	0
*Tricharina*	0.22 ± 0.36	0.02 ± 0	0.36 ± 0.45	0.08 ± 0.03	0
*Tricholoma*	0.62 ± 0.37	0.02 ± 0.04	0.06 ± 0.05	2.09 ± 3.19	0
*Tuber*	0 ± 0	0.06 ± 0.1	0 ± 0	0 ± 0	0
other	2.01 ± 1.0	9.06 ± 11.23	6.48 ± 10.28	3.07 ± 4.68	
Total					18

*Values are means ± SE (n = 3). ^a,b^Significantly different (P < 0.05) by ANOVA.*

**FIGURE 5:**
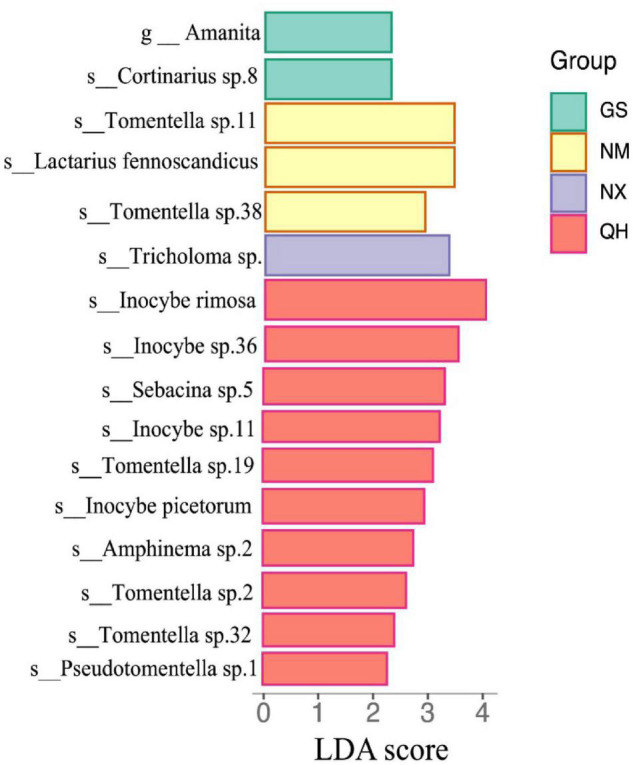
Histogram of LAD scores (LDA score > 2.0 and *P* < 0.05) for differentially abundant OTU among the four sites.

We also used LEfSe to determine which taxa most likely explained differences among locations. Each OTU with an LDA value >2 was collected with higher LDA values representing greater differences. As shown in Figure 5, only the genus *Amanita* was significantly enriched at the GS site at the genus level. Four species (genus *Inocybe*), three species (genus *Tomentella*), one species (genus *Pseudotomentella*), one species (genus *Sebacina*), and one species (genus *Amphinema*) were enriched at the QH site. Two species (genus *Tomentella*) and one species (genus *Lactarius*) showed enrichment with a high LDA score at the NM site. Both of the NX (*Tricholoma* sp.) and GS (*Cortinarius* sp.8.) sites had one dominant species.

## Discussion

Next-generation sequencing technologies have enabled large-scale analyses of the complex and normally species-rich ECM communities, showing high consistency with the clone-based Sanger sequencing (Kauserud et al., 2012). High-throughput sequencing technology has greatly facilitated the acquisition of genome data (Li et al., 2020), which allows us to investigate the ecophysiology of uncultured microbes (Wagner, 2009). In our study, the number of ECM species (196 OTUs) detected that were associated with *P. crassifolia* from the region of Inner Mongolia (NM group) is much higher than 11 OTUs at the same site (Fan et al., 2016). A total of 10 ECM species (all except 1) were found using both methods (Table 4).

**TABLE 4:** The similarity of OTU sequences between the Sanger sequencing and high-throughput amplicon sequencing associated with *P. crassifolia*.

Identified OTUs by Sanger sequencing associated with *P*. *crassifolia* (Fan et al., 2016)	Closest match by high-throughput amplicon sequencing associated with *P. crassifolia*	Sequence similarity (%)
FJ803931	OTU 10 (*Amphinema* sp.3)	97%
FJ803930	OTU 11 (*Cenococcum geophilum*)	97%
FJ803928	OTU 36 (*Cortinarius* sp.4)	100%
FJ803937	OTU 38 (*Cortinarius* sp.6)	98%
FJ803929	–	–
FJ803927	OTU 54 (*Inocybe flocculosa*)	99%
FJ803935	OTU 78 (*Inocybe* sp.9)	97%
FJ803934	OTU 131 (*Sebacina incrustans*)	100%
FJ803933	OTU 160 (*Sebacina* sp.29)	96%
FJ803936	OTU 147 (*Sebacina* sp.16)	99%
FJ803932	OTU 171 (*Suillus* sp.2)	99%

Our results clearly showed *Tomentella* was the most species-rich genus ahead of *Cortinarius*, *Inocybe*, and *Sebacina*. However, none of *Tomentella* species was detected using Sanger sequencing (Fan et al., 2016). Thus, relative to Sanger sequencing, high-throughput sequencing can capture a greater proportion of species, which is considered to be more effective in the field of community ecology (Paul et al., 2018). We found that the most species-rich ECM fungal genera were still the most common one in many other kinds of forest trees (Tedersoo et al., 2003; Richard et al., 2005; Bergemann and Garbelotto, 2006). These genera are often considered to be widespread, forming symbiotic relationships with more host plants than other ECM genera (Halling, 1987; Frøslev et al., 2005; Jakucs and Eros-Honti, 2008).

A number of studies have reported that the ECM fungal community was significantly affected by the host, soil, atmospheric deposition, geographical distance and climate (Ishida et al., 2007; Thompson et al., 2017; van der Linde et al., 2018; Wang et al., 2019). In addition to soil characteristics or even the disturbance regime of the system, the ECM fungal community structure and diversity could be determined by host trees (Johnson et al., 2005). The host age, photosynthetic activity, distributions, forest management and species composition affected the diversity or community structure of the ECM fungi (Goldmann et al., 2015; Erlandson et al., 2016; Hui et al., 2017; Unuk et al., 2019; Matsuoka et al., 2020). To explore the effects of abiotic factors, the most efficient way to eliminate the host effect is to focus on only one distributed worldwide host species such as *Pinus tabulaeformis*, *Quercus liaotungensis*, *P. crassifolia*, and *Populus simonii*. Consistent with our previous work (Long et al., 2016; Han et al., 2017; Wang et al., 2017), we found that site was the overriding factor affecting ECM fungal community structure compared with other factors at large scales (*R*^2^ = 0.37, *P* = 0.009, PERMANOVA). Most species of ECM fungi are thought to have limited distributions, which are commonly shaped by those of their hosts (Tedersoo et al., 2020). In general, the dominant ECM fungal genus associated with any specific host species is relatively consistent across the plant’s range. For instance, the dominant ECM fungal genus associated with *Coccoloba uvifera* was *Scleroderma* on a global scale (Séne et al., 2018). *Tomentella*, *Inocybe*, *Clavulina*, and *Russula* were the dominant ECM fungal genera in the *Alnus* trees (Roy et al., 2013). Consistent with our study, we found that *Inocybe*, *Sebacian*, and *Tomentella* were the dominant ECM fungal genera across the whole distribution area, accounting for more than 80% of the total relative abundance (Figure 2).

Although ECM fungal communities were dissimilar between sites (*R* = 0.33; *P* = 0.006, Anosim), the composition of the ECM fungal community was generally consistent among the four study sites. The number of overlapping species (137) far exceeds the number of unique species in each area (Figure 4B). We suggest that host identity should be the major factor that determines ECM fungal community composition. However, this finding is different from the study of the host plant *Castanopsis sieboldii* (Matsuoka et al., 2019), which suggested the explanatory power of the host was less than for climatic filtering and/or fungal dispersal. The cause of this difference could be that seawater blocked the transmission of ECM fungal spores among the whole hosts’ distribution. Furthermore, the LEfSe analysis identified 16 indicator taxa for differences among ECM fungal communities associated with *P. crassifolia* trees (Figure 5). The differences in ECM fungal community structure seem to be largely responsible for the shifts in the abundances of rare ECM fungal genera (Hui et al., 2017). Weißbecker et al. (2018) found that ECM fungal communities showed the greatest overlap of two sampled communities in a fungal functional group (approximately 80% of ECM fungal community similarity), although ECM fungal communities showed the highest pairwise community dissimilarities.

The distribution models consider the factors beyond consumable resources, such as space, to play a significant role in determining community structure (VanDerWal et al., 2009; McPolin and Kranabetter, 2021). The biogeographic position of each population on the mainland is typically classified as edge, sub edge or core (De Kort et al., 2021). QH is the core distribution of *P. crassifolia*, while other sites are at the edge in this study. ECM fungal richness decreases with the distances increasing from the core to the edge (Figure 3B), which agrees with the *abundant-center hypothesis*. Our model is similar to the non-linear model for endemic ECM species about ECM fungal community dissimilarity (based on species incidence) and interplot distance (McPolin and Kranabetter, 2021). However, unlike the pattern of ECM fungal richness distribution, the relative abundances of individual ECM fungal genera are difficult to link directly to the *abundant-center hypothesis* (Table 3). Mixed support for the *abundant-center hypothesis* has also been found in population genetic studies of other plants (e.g., Dixon et al., 2013). Furthermore, the center site (QH) has the most endemic ECM fungi (Figure 4B) and different biomarkers (Figure 5). The reason for this could be the changes in the relative abundance of dominant ECM fungi might be influenced by a specific environmental factor (Wakelin et al., 2007). For instance, nitrogen enrichment can lead to an increase in the biomass and abundance of the genus *Tomentella* (Brearley et al., 2005). In contrast, species in the genus *Sebacin*a were usually dominant in the high-phosphorus forest (Zavišić et al., 2016). In addition, the dominant genera usually belong to the cosmopolitan group, which displayed lower species turnover across the host distribution (McPolin and Kranabetter, 2021). However, most of the low relative abundance of ECM fungi, belonging to the endemic ECM group, was generally driven by environmental history as well as biological traits (Hobohm and Tucker, 2014). At a continental scale, the distributions of these ECM fungal genera may reflect dispersal and colonization limitations, as well as greater species turnover at a local scale (McPolin and Kranabetter, 2021). These forces could produce ECM fungal distributions that conform to the predictions of the *abundant-center hypothesis*.

## Conclusion

A species would achieve higher growth rates or greater stability at the core of a host species’ fundamental ecological niche than at sites farther from these optimal conditions. Thus, at core sites, hosts may have the highest diversity of ECM fungi as host effects play a critical role in the ECM fungal community assemblage and taxonomic diversity. Our results show that ECM fungal species richness was greatest at the center of the host plant’s range, consistent with the abundant-center hypothesis, while the relative abundances of individual fungal genera shifted inconsistently across the plant’s range. We encourage other researchers to test this hypothesis on other ECM fungal host species.

## Data Availability Statement

The datasets presented in this study can be found in online repositories. The names of the repository/repositories and accession number(s) can be found below: https://ddbj.nig.ac.jp, LC203765-LC205656.

## Author Contributions

QH and XW carried out the experiments. QH wrote the manuscript. Both authors contributed to the article and approved the submitted version.

## Conflict of Interest

The authors declare that the research was conducted in the absence of any commercial or financial relationships that could be construed as a potential conflict of interest.

## Publisher’s Note

All claims expressed in this article are solely those of the authors and do not necessarily represent those of their affiliated organizations, or those of the publisher, the editors and the reviewers. Any product that may be evaluated in this article, or claim that may be made by its manufacturer, is not guaranteed or endorsed by the publisher.

## Footnotes

1
http://www.gbif.org

2
http://ccb.jhu.edu/software/FLASH/

3
http://qiime.org/index.html

4
http://drive5.com/uchime/uchime_download.html

5
http://www.drive5.com/usearch/manual/uchime_algo.html

6
http://drive5.com/uparse/

7
https://blast.ncbi.nlm.nih.gov/Blast.cgi

8
http://unite.ut.ee
